# A method of deep network auto-training based on the MTPI auto-transfer learning and a reinforcement learning algorithm for vegetation detection in a dry thermal valley environment

**DOI:** 10.3389/fpls.2024.1448669

**Published:** 2025-02-13

**Authors:** Yayong Chen, Beibei Zhou, Chen Xiaopeng, Changkun Ma, Lei Cui, Feng Lei, Xiaojie Han, Linjie Chen, Shanshan Wu, Dapeng Ye

**Affiliations:** ^1^ State Key Laboratory of Eco-hydraulics in Northwest Arid Region, Xi’an University of Technology, Xi’an, China; ^2^ School of water resources and hydro-electric engineering of XUT, Xi’an University of Technology, Xi’an, China; ^3^ China Renewable Energy Engineering Institute, Beijing, China; ^4^ Central South Survey and Design Institute Group Co., Ltd., Changsha, China; ^5^ China Electric Construction Group Beijing Survey and Design Institute Co., Beijing, China; ^6^ Center for Artificial Intelligence in Agriculture, School of Future Technology, Fujian Agriculture and Forestry University, Fuzhou, China; ^7^ Fujian Key Laboratory of Agricultural Information Sensoring Technology, Fujian Agriculture and Forestry University, Fuzhou, China; ^8^ College of Mechanical and Electrical Engineering, Fujian Agriculture and Forestry University, Fuzhou, China

**Keywords:** vegetation detection, segmentation deep learning, network training automatic, data augmentation automatic, reinforcement learning for DL, auto-DL method

## Abstract

UAV image acquisition and deep learning techniques have been widely used in field hydrological monitoring to meet the increasing data volume demand and refined quality. However, manual parameter training requires trial-and-error costs (T&E), and existing auto-trainings adapt to simple datasets and network structures, which is low practicality in unstructured environments, e.g., dry thermal valley environment (DTV). Therefore, this research combined a transfer learning (MTPI, maximum transfer potential index method) and an RL (the MTSA reinforcement learning, Multi-Thompson Sampling Algorithm) in dataset auto-augmentation and networks auto-training to reduce human experience and T&E. Firstly, to maximize the iteration speed and minimize the dataset consumption, the best iteration conditions (MTPI conditions) were derived with the improved MTPI method, which shows that subsequent iterations required only 2.30% dataset and 6.31% time cost. Then, the MTSA was improved under MTPI conditions (MTSA-MTPI) to auto-augmented datasets, and the results showed a 16.0% improvement in accuracy (human error) and a 20.9% reduction in standard error (T&E cost). Finally, the MTPI-MTSA was used for four networks auto-training (e.g., FCN, Seg-Net, U-Net, and Seg-Res-Net 50) and showed that the best Seg-Res-Net 50 gained 95.2% WPA (accuracy) and 90.9% WIoU. This study provided an effective auto-training method for complex vegetation information collection, which provides a reference for reducing the manual intervention of deep learning.

## Highlights

- The MTPI method was improved to auto gain best transfer learning conditions.
- A reinforcement learning algorithm under MTPI (MTPI-MTSA) was proposed for dataset auto-augmented.
- Four deep vegetation detection networks were auto-trained with the MTPI-MTSA.

## 1 Introduction

Vegetation detection with DL (deep learning) in field UAVS [Unmanned Aerial Vehicle System(s)] images is an important fundamental technology, which has been widely used in regional hydrological information monitoring (Ang and Seng, 2021; Yang et al., 2022). Although (DL) networks, simulating animal neural structures [e.g., human or mammalian visual systems (Hassabis et al., 2017)], can accurately extract image features for (semantic) segmentation after training iterations, the training process may require lots of human experience and T&E cost (Li et al., 2020). In particular, those vegetation detection networks in unstructured environments [e.g., DTV (dry thermal valley)], which have numerous network layers, complex structures, convolutional kernels, and weighting/bias values (Saleem et al., 2021; Gentilhomme et al., 2023). Therefore, many scholars have been focusing on the DL practicality and effectiveness, such as network structure improvement (Lin et al., 2023; Liu et al., 2023; Towfek and Khodadadi, 2023), data improvement (Salcedo-Sanz et al., 2020; Kamarudin et al., 2021; Li et al., 2021), and operation methods (Al-Hyari and Abu-Faraj, 2022).

The network structure determines the functional implementation and affects the overall feature extraction ability, generalization ability, computational efficiency, and parameter number (Niu et al., 2021). So, network optimization enhances the network accuracy, improves the training effect, reduces the training iterations, and reduces the labor experience and T&E costs in training process (Shrestha and Mahmood, 2019). For example, Ouyang, S. (Ouyang et al., 2024) developed an LSBP-net structure, which incorporated a U-Net structure to reduce the vegetation influence on the lithological spectral characteristics of optical remote sensing, and the result showed a 13.94% accuracy improvement. Zhao, S.Y. (Zhao et al., 2021), Wang, P. (Wang et al., 2021) and Yang, L. (Yang et al., 2023) pointed out that adding an attention mechanism, which mimics a human vision or cognitive focus, can enhance network performance. The attention mechanisms might enhance the network performance by increasing the task-related information weight through selective reinforcement mechanisms (Ni et al., 2023). In addition, LSTM (Long Short-Term Memory) structure that mimics biological memory (Xia et al., 2020; Chen X. et al., 2023), PPM (Pyramid Pooling Module) structure (Lu et al., 2021; Pu et al., 2022), and AE (autoencoder or Self-Encoder) structure (Yang et al., 2019; Yu et al., 2023) are also common methods, when they reduce the gradient vanishing problem and improve the training effect by improving the patterns and information transmission pathways (Hu et al., 2022). However, optimizing the network structure still requires a great deal of human experience and T&E (Xu et al., 2023) in complex field environmental dataset conditions.

On the other hand, the data improvement can increase the network training rate, thus it can increase the validity of the dataset to improve the network accuracy (Nikolados et al., 2022) and reduce the time consumption (García et al., 2016). Firstly, a higher validity dataset can make it easier for the network to learn the desired features, and secondly, a valid dataset can reduce the dataset size (Ali et al., 2021), which reduces the DL training time (Rosende et al., 2023). However, when in unstructured environments, many researchers fusing on multiple data sources (Gao et al., 2020) in nowadays. Including the multi-scale same data type (García et al., 2016), multi-source data in the same form (Ding et al., 2022), and multiple data (Wang Y. et al., 2020). For example, Wei, D. P. (Weit et al., 2022) and Marzougui, A. (Marzougui et al., 2023) used multi-scale images to detect the vegetation health status, and the fusion scale method showed better performance than the traditional detection. Similarly, DL is available for fusion of different data sources, e.g., Mu, C. H. (Mu et al., 2020) used fused information from imagery and hyperspectral data, Maimaitijiang, M. (Fritschi, 2020) used fused satellite and UAV data, and Kang and Wang (Kang and Wang, 2023) used fusion multispectral and SAR (Synthetic Aperture Radar) data. In addition, DL for fusing multimodal methods is also rapidly developing field in recent years, e.g., Patil, R. R. (Patil. and Kumar, 2022) proposed a CNN network fusing meteorological and image data, Nasir, I. M. (Nasir et al., 2021) proposed a VGG 19 fusing temperature, wind speed acquired by IoT (Internet of Things), and image information, and Zheng, W. Q. (Zheng et al., 2022) proposed a Point-Net with RGB (Red/Green/Blue) images and LiDAR (Light Detection and Ranging) data. Although data fusion methods complement the network features in different dimensions to improve their performance, the data accumulation process and the data validity verification process require a lot of T&E cost and human experience.

The DL automated method implementation represents an effective approach to reducing T&E cost, and human experience. For example, the auto-PyTorch framework introduced by Zimmer, L. (Zimmer et al., 2021), the cloud auto-ML (automated Machine Learning) platform introduced by Santu, S. K. (Santu et al., 2021), and the auto-CVE system introduced by David, R. (Reyer, 2022) have been identified as effective development platforms that shorten training time, and reduced T&E. But, the simplicity necessity and performance requirements of platform constrain the DL flexibility and efficacy in complex vegetation environments. In addition, auto-DL based on intelligent algorithms could be an effective approach with minimal human intervention and T&E costs, as exemplified by the genetic algorithm proposed by Srivastava, A. (Srivastava et al., 2021), and Xiao, X. L. (Xiao et al., 2020). Furthermore, the swarm algorithm (Yeh et al., 2021), auto-stopping genetic algorithm (Montes et al., 2023), and evolutionary algorithm (Al-Hyari and Abu-Faraj, 2022) can be employed to reduce the necessity for human intervention and T&E. However, these existing auto methods involve relatively simple network structures and datasets (need to improve the practicality of those auto methods), for instance, Sun, Y. (Sun et al., 2020) designed an auto method for 10-layer networks. In conclusion, his study has proposed an automated training process for dataset augmentation and deep vegetation detection networks using transfer learning (Chen Y. et al., 2023) and reinforcement learning algorithms. Even in the complex vegetation of DTV, the proposed auto-training method requires no human intervention, ensuring network versatility and practicality.

## 2 Materials

### 2.1 Data collection and dataset

**Figure 1:**
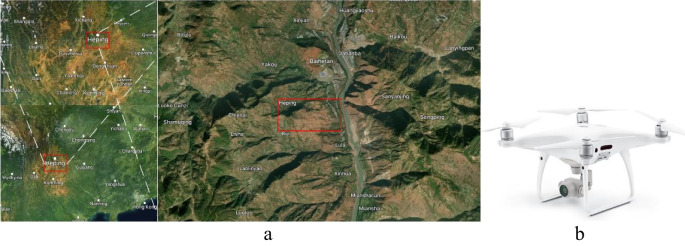
Data collection site in map and the UAVS. **(A)** the data collection environment (region marked by red rectangle). **(B)** the Phantom 4 v2 UAV.

Data collection was carried out in June 2021 near Qiaojia County, Yunnan Province, China (east 102° 53’ 11”, north 11° 9’ 10”, as **Figure 1A**), on a sunny, windless day, with steady humidity and barometric pressure. A small consumer-level 4-rotor UAVS, Phantom 4 v2 (DJI Innovation Technology Co., Shenzhen, China, as shown in **Figure 1B**), was used for the data collection. The CMOS (Complementary Metal Oxide Semiconductor) sensor of the UAVS camera is 4800×6400 p² (pixels×pixels), the lens is 3.5 mm and the image resolution is 43.4 mm². During image acquisition, the UAV flight altitude was 200 m, and the control mode was automatic control + manual model. The data collection consisted of 300 images with random occurrences in each image including mountains, buildings, water, trees, shrubs, grass, and sand.

During data collection, the UAV was operated by path planning and automatic flight control, and the remote-control system was an Android smartphone (HUAWEI P20 PRO, Huawei, Shenzhen, China) + DJI Pilot APP. The remote control was equipped with an “ON/OFF” switch, which could be switched to a manual remote controller + human judgment in the case of emergencies (encountering bird flying objects or high-altitude obstacles). **Table 1** shows the parameters set by the UAV system during data acquisition, including positioning accuracy, UAV parameters, and camera parameters (RGB camera).

**Table 1:** The parameters of UAVS for data acquisition.

UAVS	Phantom 4 v2
Lens FOV	84
Equivalent focal length	35 mm
Positioning mode	GPS/GLONASS
Vertical positioning accuracy	± 0.1 m
Horizontal positioning accuracy	± 0.3 m
Image acquisition mode	no hover shooting
Resolution of location	Less than 50 mm
Flight altitude	<500 m
Flight speed	6 m/s
Aperture	f/2.8–f/11
Camera model	FCS400
Shutter	1/2000
Resolution	4800 × 6400
ISO range	100–12,800
Photo format	JPEG/DNG*

*Only JPEG was used in this study.

**Figure 2:**
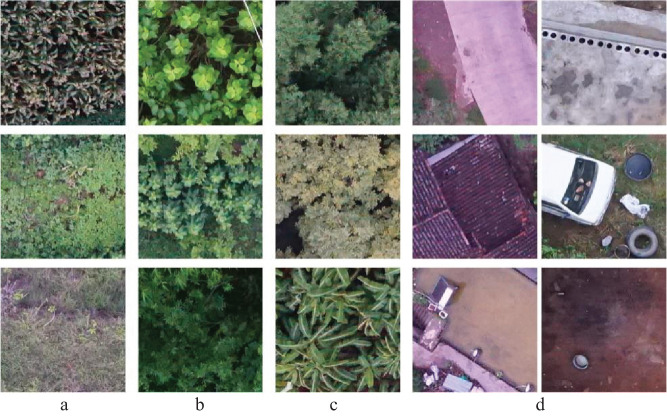
Several sample images. **(A)** random samples of pure GR. **(B)** pure SR. **(C)** pure TR. **(D)** pure NVR.

All the 300 UAV-acquired field images were split into 30,000 subregions of size 250×250 p^2^, noted as $im_{i}$ , (i=1,2,…,M) ; M=30,000 . The image content consists of various vegetated and non-vegetated elements including four categories, TR (Tree Region), SR (Shrub Region), GR (Grass Region), and NVR (Non-Vegetation Region), where the NVR may appear as buildings, water, roads, rocky, sandy, etc. The pixel regions of the dataset samples were manually labeled to distinguish different classes, such as TR (blue, (0,0,255), meaning RGB= (0,0,255), RGB means Red/Green/Blue channel values), SR [green, (0,255,0)], GR [red, (255,0,0)], and NVR [magenta, (255,0,255)], notated as $lab_{i},(i\;=\;1,2,\ldots ,M)$ . All the obtained image examples $im_{i}$ and labels mapping $lab_{i}$ were correspondingly combined into a dataset (*D*, $D\;=\{im_{i},lab_{i}\}$ ), and **Figure 2** shows several random samples of $im_{i}$ in *D*. The data in *D* was randomly divided into three parts (rounding up to the nearest integer) by 6:2:2 for networks training, validation, and testing, denoted as the training set (*TD*), validating set (*VD*), and testing set (*SD*).

### 2.2 The four deep networks and five evaluating parameters

**Figure 3:**
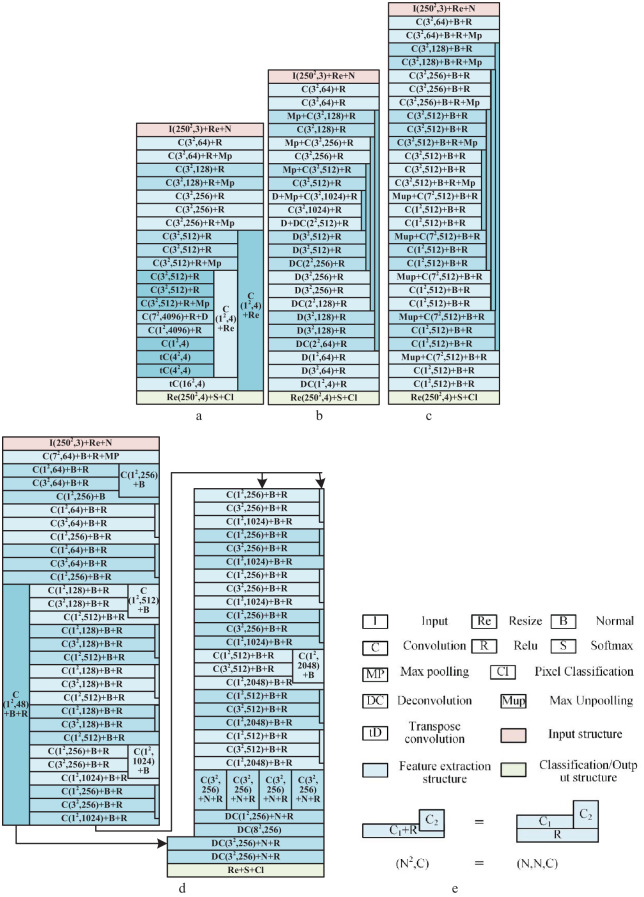
The four deep networks for vegetation detection in DTV. **(A)** The structure of FCN. **(B)** The structure of Seg-Net. **(C)** The structure of U-Net. **(D)** The structure of Seg-Res-Net 50. **(E)** Legends of networks. The size of each network layer in networks was labeled as (N^2^, C) or (N, N, C), where the (N^2^, C) means the layer filter size equivalent to N×N×C. The C is the convolution layer, DC means the deconvolution layer, MP denotes the max pooling layer, B denotes the batch normalization layer, S is the SoftMax layer, R is the relu activation layer, and Re means the resize or scaling layer.

**Figure 3** gives the four common deep network structures used in this study, including Seg-Net (He et al., 2015; Badrinarayanan et al., 2017), FCN (Long et al., 2015), U-Net (Ronneberger et al., 2015), and Seg-Res-Net 50 (He et al., 2016; Chen et al., 2018) (the resnet 50 for segmentation). Among them, FCN is a (semantic) segmentation network with a full convolutional structure proposed by Long, J., which is fast and has less memory consumption. The Seg-Net is a convolutional neural network with encoder-decoder structures, which provides high resolution and detail preservation capability when processing large-scale images or increasing the layer depth. The U-Net is a network structure with U-shape down-sampling and up-sampling, which is simple and effective. The Seg-Res-Net 50 (Chen et al., 2018) is a high accuracy and few parameters segmentation network using residual blocks, obtained by further modification of the classified Res-Net 50 (Mandal et al., 2021). Compared with the Res-Net 50, it contains additional components used to implement pixel processing capabilities, such as the inverse convolution part and the jump-joining part in **Figure 3D**.

In this study, the runtime environment used to construct, train, optimize and evaluate the network was MATLAB 2023b, running on a PC with Win10, equipped with a 24GB NVIDIA GeForce RTX 4090 GPU (graphics card) (12GHz), a 12th generation Intel (R) Core (TM) i5-12600KF CPU (3.70 GHz) and 64GB DDR4 RAM (3600MHz).

To evaluate the performance of the four networks, five parameters were evaluated in this study, including WPA (Weighted Pixel Accuracy), WPP (Weighted Pixel Precision), WPR (Weighted Pixel Recall), WPF1 (Weighted Pixel F1 Score), and WIoU (Weighted Intersection over Union). Among them, WPA characterizes the totally correctness effect of pixel detection, as shown in (Equation 1).


(1) $$\begin{array}{c} WPA=\sum_{i=1}^{C}(p_{i}\times acc_{i})\\ =1/C\sum_{i=1}^{C}p_{i}\times (TP_{i}+TN_{i})/(TP_{i}+FP_{i}+TN_{i}+FN_{i}) \end{array}$$

Where, *i* is the type of vegetation and i = 1,2,…,C (C=4). *TP_i_* is the number of true positive pixels, *TN_i_* is the number of true negative pixels, *FP_i_* is the number of false positive pixels, and *FN_i_* is the number of false negative pixels. *p_i_* is the probability that the i-th planted pixel accounts for the overall dataset images as shown in (Equation 2).


(2) $$p_{i}=\sum_{i=1}^{M}|\{C_{c}\}{|}_{i}/\sum_{j=1}^{C}\sum_{i=1}^{M}|\{C_{c}\}{|}_{i}$$

when $|\{C_{c}\}{|}_{i}$ means the total number of pixel eligible for the i-th image in the dataset (e.g., dataset D). *j* means the *j*-th vegetation types (C= 4). The WPP characterizes the correctness effect of pixel detection, as shown in (Equation 3).


(3) $$\begin{array}{c} WPP=\sum_{i=1}^{C}(p_{i}\times precision_{i})\\ =1/C\sum_{i=1}^{C}\;p_{i}\times (TP_{i})/(TP_{i}+FP_{i}) \end{array}$$

The WPR characterizes the ability of networks to find all positive pixels, as (Equation 4).


(4) $$WPR=\sum_{i=1}^{C}(p_{i}\times recall_{i})=\sum_{i=1}^{C}\;p_{i}\times TP_{i}/(TP_{i}+TN_{i})$$

And the WPF1 is the harmonic mean of precision and recall ( 0≤WF1≤1 ), with higher values showing better method performance. When the precision and recall of the method are both high, the WF1 achieves a maximum value of 1. Therefore, the WF1 is a metric that combines the precision and recall of networks and can be used to evaluate the overall network performance, expressed as (Equation 5).


(5) $$\begin{array}{c} WPF1=2\sum_{i=1}^{C}\;\\ p_{i}\times (precision_{i}\times recall_{i})/(precision_{i}+recall_{i}) \end{array}$$

The *WIoU* is the intersection over union metric, which measures the overlap between the predicted and ground truth masks, expressed as (Equation 6).


(6) $$WIoU=\sum_{i=1}^{C}\;p_{i}\times TP_{i}/(TP_{i}+FP_{i}+FN_{i})$$

## 3 Methods

**Figure 4:**
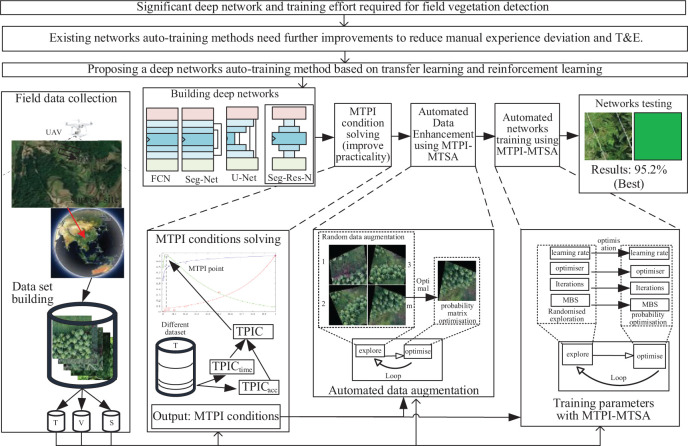
The flowchart of networks auto-training.

The **Figure 4** shows the main auto-training method process presented in this manuscript consists of three main parts, including the MTPI conditions derivation, auto dataset augmentation with MTPI-MTSA (multi-TSA based on the MTPI method), and auto network training with MTPI-MTSA.

### 3.1 Optimization of the MTPI transfer learning for deep networks auto-training

Since DL training is time-consuming and re-training of automatic methods multiplies the time consumption, it is necessary to reduce the training time to ensure automation usefulness. So, the MTPI (Chen Y. et al., 2023) (Maximum Transfer Potential Index method), which was proposed in the previous research work was improved and used in this study, to minimize the manual experience bias in complex field environments. The MTPI implements a comprehensive evaluation of the potential for a network training (TPI, Transfer Potential Index) index in both accuracy and time consumption. The minimum dataset and iteration times would be determined by obtaining the maximum TPI corresponding to the transfer training condition under low overfitting risk.

The MTPI conditions [dataset and time, or TDS and LT (learning time)] determining was obtained by maximizing the quotient of the TPI*_acc_* (Accuracy Transferable Index) and the TPI*_time_* (Time Transferable Index), as shown in (Equation 7).


(7) $$\text{TPI }={\text{TPI}}_{acc}/{\text{TPI}}_{time}$$

In this manuscript, the MTPI has been improved so that the potential indices expressed in TPI*_acc_* and TPI*_time_* refer to the actual results of the pre-training process, whereas the original MTPI (MTPI_0_) was used to derive the TPI indices for each network layer. To obtain the MTPI conditions, TDS=0.01, 0.02, 0.05, 0.1, 0.2, 0.5, 1 (×100%) were set and the TPI*_acc_* and TPI*_time_* corresponding to TDS were derived. The relationship between TDS and TPI*_acc_* was solved by logarithmic fitting to obtain *TPIC_acc_* (TPI*_acc_* curve), and the correlation between TDS and TPI*_time_* was exponentially fitted to obtain TPIC*_time_* (TPI*_time_* curve). Thereafter, the *TPIC* (TPI curve) was obtained from TDS in the range of 0 to 1 by using (Equation 7) and the corresponding training conditions (including TDS and LT) for the maximum value of TPIC (MTPI) were obtained for the subsequent automatic training process based on the MTPI.

In this study, only the vegetation segmentation problem in the DTV environment was been discussed, so the structural complexity and task complexity of the network would be constant. Therefore, it can be assumed that the size of LT is related to TDS and TPI*_acc_* is expressed as (Equation 8).


(8) $${\text{TPI}}_{acc}=\;WPA(TDS)$$

Where TDS is the migrated dataset size and WPA is the previously mentioned accuracy obtained through (Equation 3). In addition, TPI*_time_* is expressed as the relative number of iterations corresponding to TPI*_acc_*.

### 3.2 The MTPI-based reinforcement learning for dataset auto-augmentation

Data augmentation techniques (Shorten and Khoshgoftaar, 2019) is a common methods to improve the data effectiveness and reduce the overfitting risk for network training, such as Abayomi-Alli, O.O. (Abayomi-Alli et al., 2021), Ottoni, A.L.C. (Ottoni et al., 2023), and Ayhan, B. (Ayhan et al., 2020) pointed out. However, the processes and range parameters they used in data augmentation were obtained based on manual experience, and such inclusion of human intervention might easily introduce cognitive biases and manual errors. For this reason, we combined the previously described MTPI and reinforcement learning (Watkins and Dayan, 1992; Russo et al., 2018) [e.g., TSA (Russo et al., 2018; Dai et al., 2022)] to automatically determine the optimal processing methods and parameter ranges in the data augmentation process. The TSA is a common reinforcement learning algorithm for solving the optimal strategy problem for intelligence in DTV.

Since the data auto-augmentation process involves two-dimensional strategy problems with different processing method processes (M) and different value ranges (N), the traditional TSA was improved to MTSA (Multi-Thompson Sampling algorithm) with multiple levels of inputs for implementation (M×N). Where the inputs can be represented as an input matrix *Q*, as represented in (Equation 9).


(9) $$Q=[Q_{1},\ldots ,Q_{M}]=[\begin{array}{ccc} q_{1,1} & \ldots & q_{M,1}\\ \vdots & q_{n,m} & \vdots \\ q_{1,N} & \ldots & q_{M,N} \end{array}]\;$$

where, *m* is the number of augmentations method, with *m =* 1, …, M , e.g., M = 10 for this study. Where *m* = 1 denotes a random reduction with a magnification of 0 to 1, *m* = 2 denotes a random enlargement with a magnification of larger than 1, *m* = 3 denotes an inverse rotation with a rotation angle of less than 0, *m* = 4 denotes a forward rotation with a rotation angle of larger than 0, *m* = 5 denotes left-biased horizontal cropping, *m* = 6 denotes right-biased horizontal cropping, *m* = 7 denotes vertical cropping in the downward direction, *m* = 8 denotes vertical cropping in the upward direction, *m* = 9 denotes horizontal translation in the left direction, and *m* = 10 denotes horizontal translation in the right direction. *N* is the value range of each column matrix $Q_{m}={\left[q_{1},\ldots ,q_{N}\right]}_{m}^{T}$ which is evenly partitioned into *N* value accuracies, in this study, it is assumed to use a variable with 8-bit storage space to represent the value possibilities, thus N=256 (denoted as n=1,2,…,256 or n=0,1,2,…,255 ). In MTSA, the probability of each column matrix $Q_{m}$ is calculated separately. It is denoted as (Equation 10).


(10) $$P(q_{n,m})=q_{m}/\sum Q_{m}$$

Before iteration, all elements of *Q_m_* were set to 1 to avoid a zero denominator. During each iteration, the corresponding element of *Q_m_* that achieves the maximum result will be updated to bias the MTSA results towards achieving the best value, as (Equation 11).


(11) $$q_{n,m}^{t+1}=\;q_{n,m}^{t}+r\times \text{log}\;(\text{t})$$

Where r is the learning rate, e.g., r = 4 . The *t* is the number of MTSA iterations. In addition, $q_{n,m}$ also sets upper and lower limits to prevent errors due to overflow of computer digits, e.g., $10^{-10}$ and $10^{10}$ . With Q renewing, the data augmentation process and the values ranges will move towards biasing the optimal feedback (the network accuracy) to obtain the optimal solution (or near-optimal solution).

Because of the manual data augmentation methods usually use the maximum processing and commonly available parameters (e.g., multiples of 10). Therefore, to gain simulated manual method results for comparison, all the involved processing and five common parameters were taken for data augmentation, including *mt*  = −5,−4,…, 5 . During the test of data augmentation, mt would be mapped to actual values. Where, the reduction is $\left(1-10^{mt}\right)$ , the enlargement is $1/(1-10^{mt})$ , the rotation angle is $\pm [45\times 10^{mt}]$ , and the offset or crop value is $\pm [50\times 10^{mt}]$ , the [ ] means rounding up to the nearest integer.

**Figure 5:**
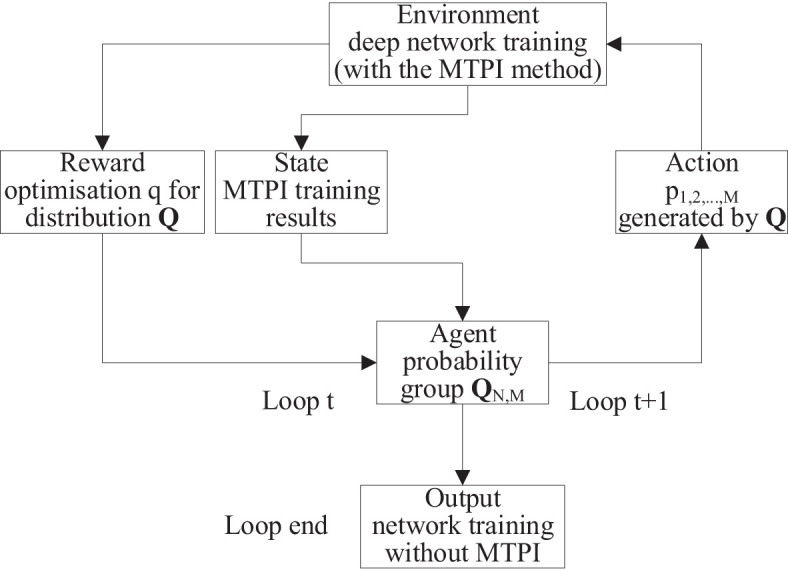
The block diagram of MTSA reinforcement learning.

**Figure 5** shows the schematic of MTSA reinforcement learning in this paper, including the interactions of the agent, environment, state, action, reward, and output. The agent performs a t+1 action according to the policy (Equation 11) based on the t state and the t reward feedback from the environment. The Agent is a hypothetical individual acting in the environment, and the actions it produces in the environment are controlled by a probability distribution Q that can be iteratively optimized. In the t+1 loop, the t+1 action of the agent consists of M sub-actions of $p_{1,2,\ldots M}$ . The action $p_{1,2,\ldots \text{M}}$ is transitioned to a t+1 state in the environment, and a t+1 reward positive or negative reward) is generated. The output of the environment (means the agent in environment) is the MTPI training result corresponding to the action, and although each action is retrained in multiple cycles, the time consumption of the MTPI has been significantly reduced, and the whole reinforcement learning process is still acceptable. After the maximum number of loops is reached, the result output by the agent is used as the auto-training parameter of the network (the output result is without MTPI).

### 3.3 The MTPI based reinforcement learning for deep network auto-training

The MTPI-MTSA introduced in **section 3.2** is an effective automatic method to replace manual experience, so it is similarly used in the DL auto-training. In the implementation of MTPI-MTSA for network auto-training, the mapping form of the input matrix was optimized to make the inputs match the auto-training. The cost function of MTPI-MTSA was also optimized so that the output considers the result accuracy and the time consumption factor would not be ignored at the same time.

As reported in scholarly studies, the training parameters affecting the DL accuracy and time consumption include, OT (Optimizer Types) (Elshamy et al., 2023), LR (Learning Rate) (Lewkowycz et al., 2020), IN (Iterations Number) (Xiang et al., 2021), and MBS (Mini Batch Size) (Kandel and Castelli, 2020). Therefore, the input matrix Q of the MTPI-MTSA contains the four-parameter dimensions mentioned, denoted as M=4 and N=256 to represent them during the auto-training. Among them, m=1 represents the OT, and the optimizer introduced in this paper included “sgdm” (Stochastic Gradient Descent with Momentum), “Adam” (Adaptive Moment Estimation), or “RMSprop” (Root Mean Square Propagation) which was shown as (Equation 12).


(12) $$OP=\{\begin{array}{c} \begin{array}{cc} 1 & ,q_{1}<N/3 \end{array}\\ \begin{array}{cc} 2 & ,N/3\leq q_{1}<2N/3 \end{array}\\ \begin{array}{cc} 3 & ,2N/3\leq q_{1} \end{array} \end{array}$$

Where *m* is the ordinal number of $q_{m}$ , OP=1 means the OT is “sgdm” optimizer, OP=2 means the “Adam” optimizer, and OP=3 means the “RMSprop” optimizer. In addition, m=2 denotes the LR and the mapping relationship was given in (Equation 13).


(13) $$LR=10^{-1\times (l+r\times q_{2}/N)}$$

Where *l* and *r* are the two limiting factors of the LR value range, such as l=1 and r=6 . Thus, the value range of LR is $\left[10^{-7},10^{-1}\right)$ . And then m=3 denoted the max epochs of IN, and it will be rounded to the nearest integer. When m = 4 , the MBS with the value range (1,256), its upper bound can be changed according to the GPU RAM, but must be an integer.

To improve the MTPI-MTSA on network accuracy and time consumption to make deep training automatic, a time consumption component is added to the cost function, as shown in (Equation 14).


(14) CF=A×t/T 

Where *A* is the accuracy, *T* is the relative iteration number (relative time consumption), and t is the iteration number where the minimum loss was obtained in the training process. Since *A* has a higher priority relative to *T*, the *T* will be considered only when $A\geq A_{0}$ , while the $A_{0}$ can be set as the MTPI result in section 3.1 and section 3.2. Where *T* can be obtained as (Equation 15).


(15) $$T=t/t_{0}\times 100\%$$

To further validate and test the effectiveness of the complete network auto-training, the data auto-augmentation and network auto-training were performed on the structures of FCN, Seg-Net, U-Net, and Seg-Res-Net 50 built-in section 2.2. Where the data auto-augmentation was performed in the MTPI-MTSA method introduced in section 3.2 and section 4.2 as *AD*. The four networks were auto-trained with the methodology presented above and were tested with the *SD* introduced in section 2.1.

## 4 Results and analysis

### 4.1 Results of the improvement MTPI for deep learning auto-training

**Figure 6:**
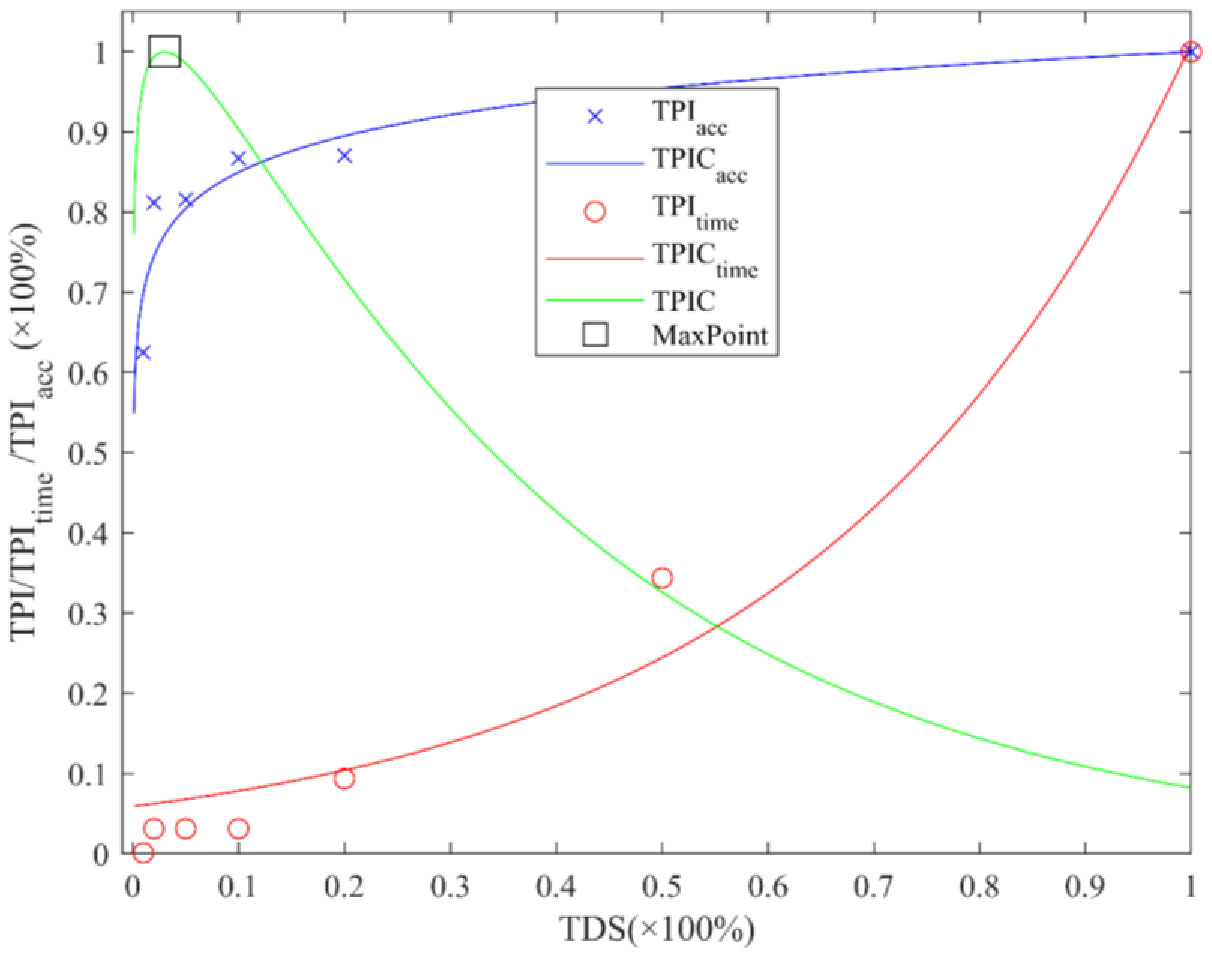
Results of network auto-training conditions determined by the improved MTPI.

The **Figure 6** shows the results of network auto-training conditions (the MTPI conditions) determined by the improved MTPI in DTV environment. The graph, including the TPI*_acc_* index (when TDS = 0.01, 0.02, 0.05, 0.1, 0.2, 0.5, 1 ), the TPI*_time_*, the fitting curve of TPIC*_acc_* [when TDS∈(0, 1) ], the fitting curve of TPIC*_time_*, the TPIC index curve, and the value of MTPI (the maximum point), shows the impact of the TDS value on the network accuracy and time cost. The trends of TPIC*_acc_* and TPIC*_time_* showed that the potential indices of accuracy and time spent would both increase as the TDS increases; but the rising trend of TPIC*_acc_* keeps decreasing, while the rising trend of TPIC*_time_* keeps increasing. This might be due to the TDS influence decreasing as the network gets closer to the optimal when its training process gets more difficult; but the effect of TDS on accuracy is still controversial, (conjecture that under field vegetation data conditions TDS can be close to a logarithmic relationship with the network performance). In addition, the relationship of TDS on TPIC*_time_* (training time consumption) is approximately exponential, which may be due to TDS increase affecting not only the batch number but also the final network performance when training in MBS (mini-batches size). The TPIC trend is increasing and then decreasing which is consistent with the trends of TPIC*_acc_* and TPIC*_time_* analyzed earlier, which might be because TPIC*_acc_* (the numerator of the TPI obtaining formulae) increases faster when TDS is small whereas TPIC*_time_* (the denominator of the TPI) increases slowly; thereafter, the TPIC*_acc_* increases at a reduced rate and TPIC*_time_* increases at an elevated rate. It also shows that, when the TDS is small, an increase in TDS can quickly improve the network performance without much change in time consumption. However, when the TDS is already large, a further increase in TDS has less impact on the network performance but consumes a huge amount of time, which is consistent with the phenomenon that the performance enhancement of DL slows down gradually in network training. The value corresponding to the TPIC maximum (maximal point) is the result of the MTPI method (MTPI condition, including TDS and LT). Obviously, the MTPI condition is not the performance-optimal condition for networks, but this condition is the best-combined combination of performance and time cost, which is beneficial to discuss the improvement of DL and optimization of parameters.

More specifically, the fitted curve ${\text{TPIC}}_{acc}$ has the eq. ${\text{TPIC}}_{acc}=a\times \text{ln}(TDS)+b$ , where a = 0.0650 and b = 0.995 , the RMSE =0.0485 and the $R^{2}=\;0.858$ . The fitted equation for the fitted curve ${\text{TPIC}}_{time}$ is ${\text{TPIC}}_{time}=a\times e^{\left(b\times TDS\right)}$ , where a = 0.0591 and b = 2.84 ; the RMSE =0.0604 and $R^{2}\;=0.977$ . Thus, the MTPI conditions obtained are TDS = 2.30% , LT = 6.31% , and the reference accuracy $WPA_{ref}\;=\;76.3\%$ .

It can be seen that with the introduction of the MTPI method, the optimization process requires only 2.30% dataset size and 6.31% time-consuming. The MTPI has largely improved the utility and adaptability for later network auto-training. In this study, we investigate the transfer training results of the same network [trained in a detection task (He et al., 2016)] transferred to the same dataset with different parameter conditions, while the previous paper investigated the transfer training of a network on a different dataset, but we considered such results still reasonable. The reason might be that the starting point of TL (He et al., 2016) has obtained some basic primitive features (e.g., edges or corner points), and all these features have a strong role in the detection of the image. However, as can be seen from the starting point of the curve in the figure (e.g., TDS < 0.05), the network without TL has a relatively large number of non-universal features (lower accuracy, <0.6). This result also reflects, to a certain extent, the superiority of the MTPI algorithm, which aims to achieve speedups under the premise of obtaining the most efficient results with the smallest TDS.

### 4.2 The results of auto dataset augmentation with MTPI-MTSA

**Figure 7:**
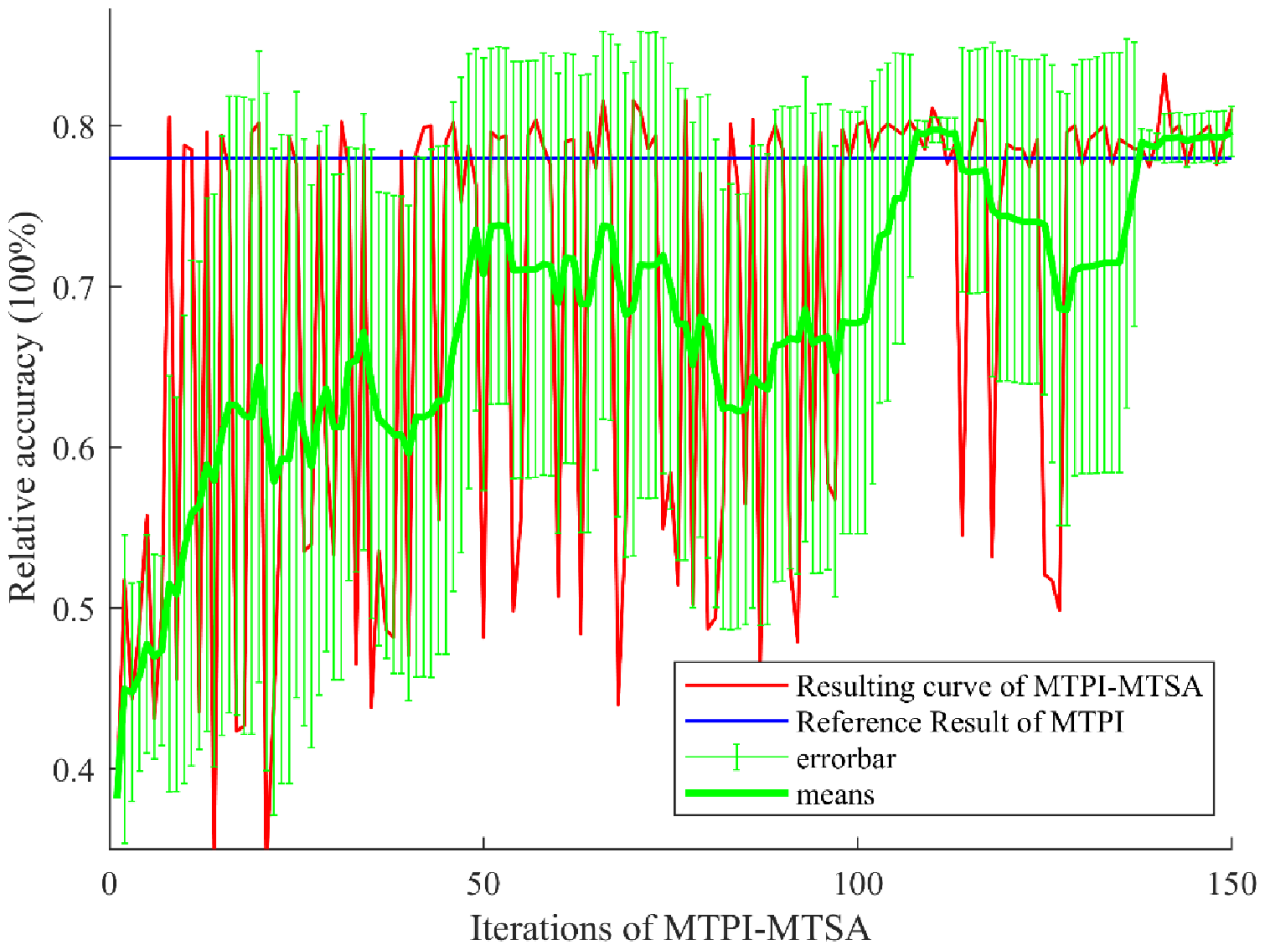
MTPI-MTSA Results of the automated augmentation dataset.

**Figure 7** gives the output curve as iterations of the MTPI-MTSA during the dataset auto-augmentation of the optimal solution. This contains the red result curve (accuracy, WPP), the blue reference curve (obtained from the MTPI method above), the green 10 t (times of iteration) average, and the 10-t mean-error curve (10 t standard deviation of the green curve). The red curve shows that the algorithm gets poor (<55%) results in the early stage (≤50 t), changes significantly in the middle stage (>50 and ≤100 t), and shows a high result and small changes in the later stage (>100 t). This resultant performance corresponds to the mean-error curve, with low mean scores and low errors in the early part, then the mean scores increase in mean but with higher errors in the middle part, and the mean scores stabilize at a larger value (compared to the reference line) with low errors in the late part. These results are also consistent with the TSA which has phases of self-exploration and convergence to the best value. The MTPI-MTSA falls back near the later stages (e.g., 110-130 t periods), which may be due to possible unfavorable exploration in the MTSA iterations, but stabilizes at a higher level in the later stages as the algorithm converges towards better results, which may be due to the late algorithmic effect of the algorithm approaching the optimal solution. This also suggested that the relationship between data enhancement variables and network accuracy for MTPI-MTSA in field vegetation data is likely not a probability distribution in the traditional sense (e.g., normal or beta).

**Table 2** shows the data augmentation methods and parameters obtained by the proposed MTPI-MTSA, including Reduce Scale, Enlarge Scale, Rotate, Offset Left/Right, Crop Left/Right, Crop Up/Down, and Offset Up/Down. The table shows that the scale range between 0.81 and 1.64 is an optimal value (or near-optimal value), which may be related to the UAVS and DTV terrain factors. Although the UAVS is operating at 200 m altitudes, the DTV has a large difference in horizontal altitude and terrain, so it is necessary to have some image scale optimization to achieve the differentiation. In addition, the offset and cropping of the images are concentrated around 23 (between 18 and 26, which is about less than 10%, with an image scale of 250), probably because the offset and cropping have similar linear geometric transformations (concentrated in constant terms in the image transformation matrix). However, they still have some data errors, which is possible because the optimization process of MTPI-MTSA is stochastic and the error in the results is the stochastic optimization process. Such results with some errors are acceptable, firstly because the subsequent auto-training process can accept a certain range of data augmentation results, and secondly because such data augmentation with variation can lead to more diverse stochastic data for training.

**Table 2:** Results of the dataset augmentation method obtained with the MTPI-MTSA.

Methods	Lower limit	Upper limit
Reduce/Enlarge Scale	0.81	1.64
Rotate	-25.88	36.08
Offset Left/Right	–	–
Crop Left/Right	-21.96	19.80
Crop Up/Down	-25.88	18.82
Offset Up/Down	–	–

The results of the manual method show that when mt=−5, −4,…, 5 WPA = 73.05, 72.54, 73.12, 69.61, 14.56, 73.70, 73.11, 73.42, 68.54, 26.13 (%), with a mean value of 60.30% and a std. var. of 20.94 ( ref. = 76.3 (%). Comparing the manual and automated method results shows that the optimal results of the manual method can be close to the reference value, but the manual method has a T&E cost (std. > 0), which explains that the manual method needs multiple tests and parameter debugging to obtain the best. The introduced MTPI-MTSA automatically obtained the optimal results (or near-optimal solutions) with no human involvement or T&E process, which also proves that the MTPI-MTSA is necessary for fully automated data augmentation of network training. The results showed that the manual method was worse than the automated and it might be because the manual method used all processing methods and uniform parameters in this study, but not all of them were suitable for vegetation segmentation in DTV environments.

### 4.3 The auto-training results of deep networks with MTPI-MTSA

**Figure 8:**
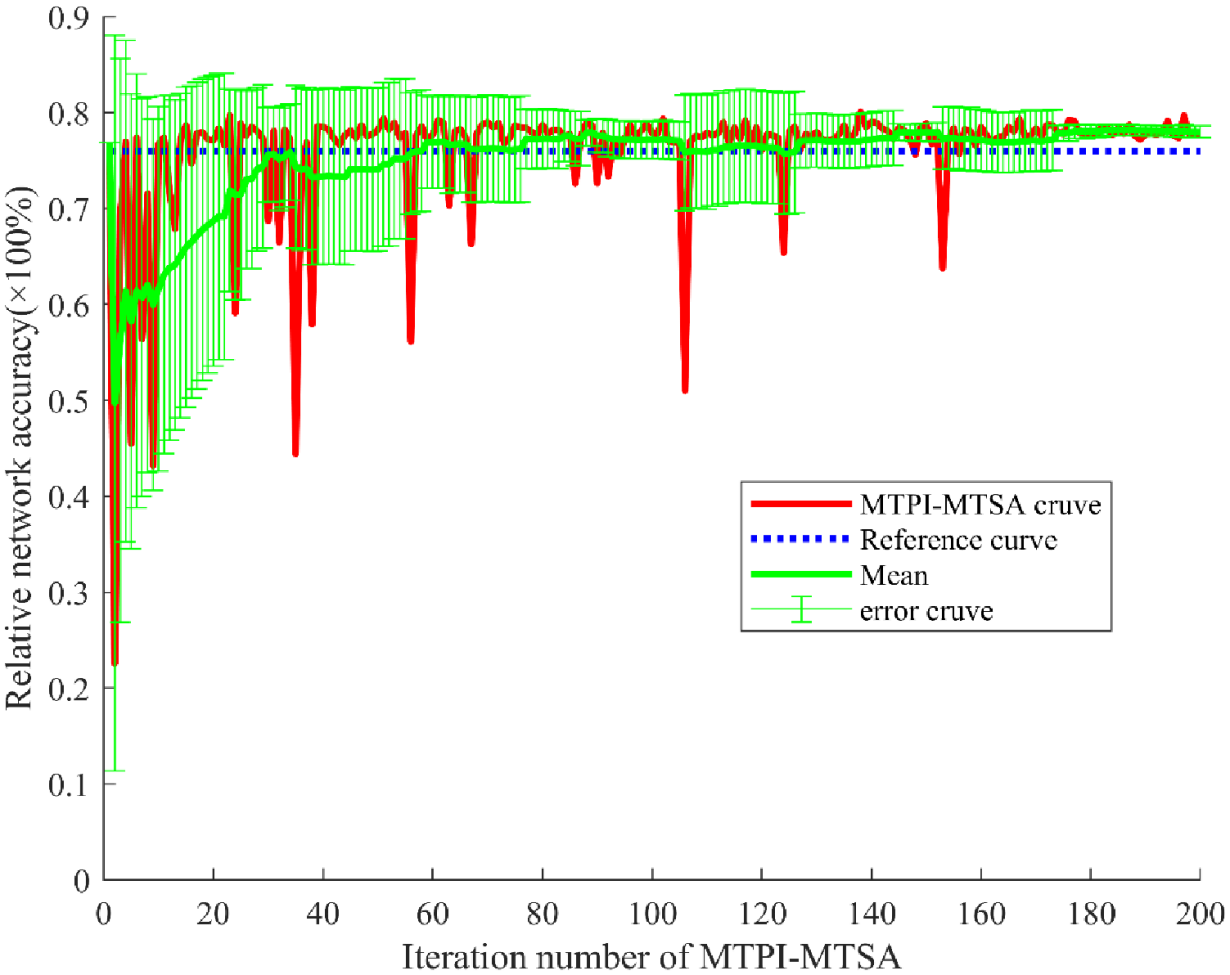
The MTPI-MTSA results of networks auto-training.

**Figure 8** shows the networks auto-training results with the MTPI-MTSA, including the iterative results curve (WPP) in red, the reference curve in blue (derived from the improved MTPI), the 10-iteration average curve and the 10-iteration mean-error curve in green (standard deviation for error). Similar to section 4.2 the iterative optimization process can be divided into three stages, a pre-stage (≤50 t), a middle stage (>50 and ≤150 t), and a late stage (>150 t). It can be seen that the resulting curve and the average curve gradually increase from the lowest point but contain a large error in the early stage, which is consistent with the TSA has a certain self-exploration and tends to the optimal law, and the results are also consistent with the results in section 4.2. In addition, the output and average curves showed a high output with a gradually decreasing error in the middle stage, which indicates the algorithm score can reach a larger score position and gradually smooth out. Although the process can still be unfavorable exploration and lead to a certain degree of fluctuation in the output curve, as the iterations increase, the probability of unfavorable exploration is gradually reduced. Finally, both the result curve and the mean curve are at higher curves and the error is small in the late stage. The mean value of the score increases but also has a larger error in the middle of the algorithm, and stabilizes at a larger value (above the reference line, blue line) and with a smaller error in the later stages. Such curve performance indicates auto-training, it may be due to the structure of the MTPI-MTSA used and the fact that the optimization training in the DTV environment has a more concentrated high probability of taking values (Compared with trends in data auto-augment above).

**Figure 9:**
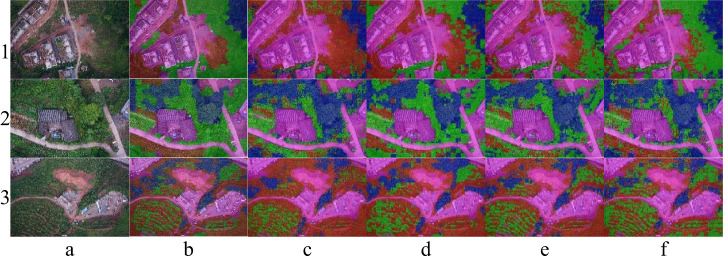
Results of the four pre-trained deep networks for the segmentation of four samples. **(A)** RGB image. **(B)** Reference result image. **(C)** Results of a-image processing using Seg-Net. **(D)** Results of a-image processing using FCN. **(E)** Results of a-image processing using U-net. **(F)** Results of a-image processing using Seg-Res-Net 50.

To further demonstrate the network auto-training method proposed in this paper, the established Seg-Net, FCN, U-Net, and Seg-Res-Net 50 network structures were similarly processed and compared. Three sample images were randomly selected for processing and testing respectively, which have been given in **Figure 9**. In the labeled RGB image results, blue, red, green, and magenta markers with 20% transparency were used to indicate the segmented regions of TR, SR, GR, and NVR, respectively. Column a in each row represents the original RGB image, column b represents the manually labeled results (ref.), and columns c, d, e, and f represent the network output. Overall, the four networks achieve vegetation segmentation to some extent, e.g., all four networks achieve good results in the NVR compared to column b. It might show that the segmentation task in the NVR is the easier part, when the NVR features are visible and obvious (e.g., color and texture feature), even easy for the human eye. In addition, the U-Net seems to treat the SR part more as TR, e.g., **Figure 9** 1. e (1. e denotes the resultant image in row 1, column e), while the FCN misclassifies it more as GR, e.g., **Figure 9** 2. e, which may be related to the convolution depth of the network, with deeper networks having better resultant performance. However, Seg-Res-Net 50 still significantly outperforms U-Net in the SR region, as shown by the difference between 3. e and 3. f in **Figure 9**, which may be related to the performance structure. In these results, the segmentation of Seg-Res-Net 50 is the best among all vegetation species, which may be related to the network residual structure introduced in Seg-Res-Net 50, which is consistent with the results of Alfanindya, A. (Alfanindya et al., 2013). and Yu, H. (Yu et al., 2021).

**Figure 10:**
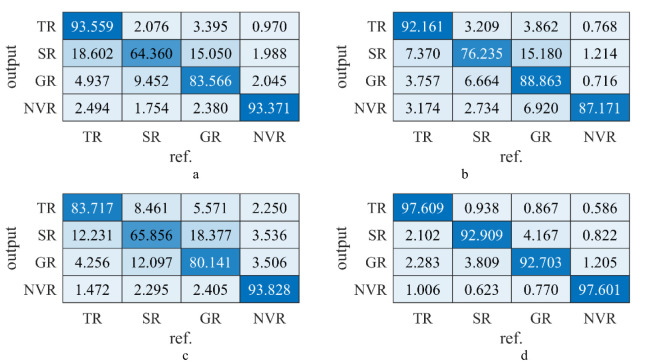
Confusion matrix results of the four auto-trained networks on the SD. **(A)** Confusion matrix of FCN. **(B)** Confusion matrix of Seg-Net. **(C)** Confusion matrix of U-Net. **(D)** Confusion matrix of Seg-Res-Net 50.

**Figure 10** shows the confusion matrix results (an overall statistical result of a dataset) for the four networks on the *SD* (testing set, introduced in section 2.1). The horizontal axis was the pixel point mapping output results for the for categories, while the vertical axis represents the actual category, i.e., the first column on the horizontal axis corresponds to the TR, SR, GR, and NVR. The diagonal elements of the confusion matrix from the top left to the bottom right reflect the network accuracies for the four categories, with higher values showing better network performance. The results showed that all four networks achieved some performance on the dataset, with Seg-Res-Net 50 achieving the highest overall accuracy (97.60% in **Figure 10D**), while Seg-Net showed the lowest overall accuracy (64.36% in (**Figure 10A**). The results in **Figure 10** showed that Seg-Net had a weak segmentation performed for SR and GR (**Figure 10A**, only about 64.36 and 83.57%), FCN is slightly better than that of Seg-Net for GR and SR (about 10% improvement on SR, **Figure 10B**), on the other hand, Seg-Res-Net 50 was better than the three previous network structures in all categories (**Figure 10D**). This result may be related to the residual connection structure in Seg-Res-Net 50 since the residual network was allowed to learn deeper structures efficiently without being prone to overfitting, which is consistent with the earlier findings.

**Table 3** shows the results of the four auto-trained networks on the SD from five perspectives (WPA, WPP, WRE, WF1, and WIoU). Among them, WPA is the overall prediction accuracy (intuitive performance); WPA is the accuracy of the positive category prediction (positive category accuracy); WRE is the coverage for the actual positive category (positive category detection); WF1 is the evaluation of the network performance (a combination of WPA and WRE); and WIoU is the overlap between the actual and predicted regions. Based on the above five evaluation perspectives, Seg-Res-Net 50 was found the best overall performance than Seg-Net, U-Net, or FCN, (e.g., the WIoU in **Table 3**). These results may have been attributed to the fused residual structure of the Seg-Res-Net 50 with deeper network layers and better vegetation segmentation capabilities. Combining the above example analysis (**Figure 10**) and the statistical results analysis (**Table 1**), the auto-trained Seg-Res-net 50 outperformed the other three networks in terms of overall segmentation effect and per-class segmentation effect. This also indicated that Seg-Res-net 50 performed well on simple tasks (NVA) and complex tasks (GR, SR, and TR), both in detail and overall. It also demonstrates that the proposed auto-training method, based on MTPI and reinforcement learning, can be used in complex network structures (e.g., Seg-Res-Net 50) and complex dataset environments (e.g., DTV in the field).

**Table 3:** Results of five evaluation factors for the four network structures (%).

Networks	WPA	WPP	WPR	WPF1	WIoU
FCN	83.714	84.023	83.714	83.395	72.283
Seg-Net	86.108	86.691	86.108	86.142	75.940
U-Net	80.886	80.704	80.886	80.706	68.463
Seg-Res-Net 50	95.205	95.201	95.205	95.195	90.881

To verify the advantages of the proposed network auto-training method in this manuscript, four similar methods are given in **Table 4** for comparative discussion. The evaluation perspectives include dataset size, data sample size, network structure, acceleration methods, and acceleration effectiveness. From the two perspectives of dataset size and sample size (the two columns of the table), the proposed method has a larger dataset, and the object of the dataset is the vegetation image data in an unstructured DTV environment, which can be seen that the proposed method has a higher degree of compatibility. In addition, the network structure in **Table 4** shows the proposed method is more adaptable to complex networks (comparing the network depth of the proposed method and the previous three methods). Although Wang, S.’s method has a more complex structure, his paper does not include the process of automatically determining the optimal training parameters, which might require the algorithm to repeatedly confirm the optimal conditions. This result also implies more manual setup experience for implementation, and it further surfaces the greater practicality of the proposed method. Finally, the acceleration methods and acceleration effects are also compared, which shows the potential future of the overall method. Among them, the acceleration means of the proposed study, MTPI, improves the overall iteration speed to a great extent, making the proposed auto-training method very promising for future applications.

**Table 4:** Performance differences compared to three existing methods.

Methods	Dataset size	Sample size	Networks	Acceleration method	Time cost
Proposed method	30,000	3×250^2^	Res-Net 50 (58) ^a^	MTPI	8.7%
Sun, Y. method (Sun et al., 2020)	6,000	3×32^2^	Auto-CNN (10)	- ^b^	100%
Xiao, X. method (Xiao et al., 2020)	6,000	3×32^2^	CNN (14)	early stop	75%
Montes, C. Method (Montes et al., 2023)	220,000	1×128	CNN (10)	early stop	50%
Wang, S. method (Wang S. et al., 2020)	1266	1×512^2^	DenseNet121(>60)	–	–

a The number in “()” means layers number, such as the “Seg-Res-Net 50 (58)” means the Seg-Res-Net 50 has 58 layers. In this paper, the network layers number is denoted by the number of active layers.

b The “-” means used no time-saving method.

## 5 Conclusion and discussion

To reduce the human intervention and T&E cost for deep network training as well as to enhance the existing methods’ practicality and adaptability, an auto-training method was proposed based on the MTPI and MTSA in the DTV environment. The main conclusions are as follows.

1. The MTPI was improved to automatically calculate the most suitable transfer conditions and found that the subsequent automation only requires 2.30% data and 6.31% time, which is adaptable to the complex networks in DTV.
2. The MTPI conditions were incorporated into the automatic dataset augmentation prior to network training and found a 20.94% T&E reduction and a 16.00% accuracy improvement.
3. The auto-training method was used for four common networks (e.g. FCN, Seg-Net, U-Net, Seg-Res-Net 50) in a DTV environment, and the best Seg-Res-Net 50 achieved 93.6% WPA and 87.6% WIoU. The proposed auto-training is more practical than several existing methods, and avoids human intervention, providing a reference for automated deep learning applications in unstructured environments.

## Data Availability

The raw data supporting the conclusions of this article will be made available by the authors, without undue reservation.
